# Effect of ethnic diversity on the saccadic reaction time among healthy Indian and Dutch adults

**DOI:** 10.1038/s41598-023-50670-8

**Published:** 2024-01-04

**Authors:** Najiya Sundus K. Meethal, Deepmala Mazumdar, Gijs Thepass, Hans G. Lemij, Johannes van der Steen, Johan J. M. Pel, Ronnie George

**Affiliations:** 1 Department of Neuroscience, Vestibular and Ocular Motor Research Group, Erasmus MC, Rotterdam, The Netherlands; 2 Medical Research Foundation, Chennai, India; 3 Rotterdam Ophthalmic Institute, Rotterdam, The Netherlands; 4 Glaucoma Service, Rotterdam Eye Hospital, Rotterdam, The Netherlands

**Keywords:** Neuroscience, Biomarkers, Diseases, Medical research

## Abstract

Eye movement perimetry (EMP) expresses the decline in visual field (VF) responsiveness based on the deviation in saccadic reaction times (SRTs) from their expected age-similar responses (normative database). Since ethnic dissimilarities tend to affect saccade parameters, we evaluated the effect of such a factor on SRT and its interaction with age, stimulus eccentricity, and intensity. 149 healthy adults, spread into five age groups, drawn from Indian and Dutch ethnicities underwent a customized EMP protocol integrated with a saccade task from which the SRTs to ‘seen’ visual stimuli were computed. The EMP test had a total of 54 coordinates (five stimulus eccentricities) tested using Goldmann size III visual stimuli presented at four stimulus intensity (SI) levels against a constant background. Considering SRT as a dependent variable, a Generalized Linear Mixed Model analysis was conducted that revealed a statistically significant (p < 0.001) influence of ethnicity and interaction between the tested factors (ethnicity × age × stimulus eccentricity × intensity). However, during the post hoc analysis, out of the 100 possible pair-wise comparisons, only 6% (minor proportion) of the estimates showed statistical significance. Hence, the ethnic-specific differences need not be accounted for while implementing EMP in a diverse set of populations instead a collective database might serve the purpose.

## Introduction

Cultural neuroscience claims ethnicity and cultural exposure to be influential factors in modelling basic cognitive behaviour in humans including the pattern of eye movements^1^. Eye movements have been widely used to assess tasks pertaining to information processing such as text reading, scene perception, face recognition, and visual search. A wide range of studies has formerly reported differences in Saccadic Eye Movement (SEM) parameters across different ethnic groups while performing saccade tasks^2–5^. Since the genetic constitution and ethnic dissimilarities are found to affect eye movement behaviour, it is recommended to consider these influential factors while relying on SEM metrics for detecting neuronal loss^5^.

On the basis of an SEM parameter, the Saccadic Reaction Time (SRT), our study group has previously reported a customised Eye Movement Perimetry (EMP) system as a viable method of perimetry in adults^6,7^ as well as in children^8^. In the adult cohort, we have reported the feasibility and clinical applicability of the system in detecting the neurodegeneration associated with glaucoma^6,7^. Our EMP protocol incorporated a saccade task integrated with an infrared-based eye tracking device (Tobii T120, Tobii, Sweden), which captured the SEM responses. We primarily focused on the SRT, defined as the measured time gap (milliseconds) between the appearance of the visual stimuli and initiation of the SEM, as an index to estimate the Visual Field (VF) responsiveness^6–8^. We have found glaucoma patients to exhibit significantly delayed SRT values when compared to their age-matched healthy controls regardless of their disease severity^6,7^. Furthermore, previous studies have also reported the occurrence of altered SEM properties considerably earlier than any evident functional defects detected using Standard Automated Perimetry (SAP)^9–11^.

In EMP, the glaucomatous functional loss is flagged based on the binary responses (seen/unseen) and deviation of SRT values from their age-expected responses^6,7^. To generate a normative database, we have previously evaluated the effect of age, gender, stimulus eccentricity, and Stimulus Intensity (SI) on SRT within the tested VF^12^. Still, an additional evaluation of SRT variability across different ethnicities seemed necessary to explore whether distinct normative databases are required while introducing the proposed EMP system in different ethnic cohorts. Hence, the current study primarily aimed to evaluate the influence of ethnic diversity among healthy adults, drawn from two different ethnic backgrounds, in combination with factors such as participants’ age, stimulus eccentricity, and intensity on SEM responses and SRTs while performing saccade tasks in EMP.

## Materials and methods

### Study participants

Participants included healthy volunteers aged between 20 to 85 years recruited at two institutes: (1) Sankara Nethralaya (a tertiary eye care centre), Chennai, India, (source of Indian ethnic cohort) and (2) Erasmus Medical Centre, and The Rotterdam Eye Hospital Rotterdam, The Netherlands, (source of Dutch ethnic cohort). The study methods adhered to the tenets of the Declaration of Helsinki and the experimental protocols were reviewed and accepted by the Institutional Review Board of Vision Research Foundation, Chennai, India, the Medical Ethics Committee of Erasmus Medical Centre, Rotterdam, and The Rotterdam eye hospital, The Netherlands. Written informed consent was obtained from all the participants before enrollment and initiation of any procedures.

Healthy participants were defined as those individuals with a best corrected visual acuity of  ≥ 20/40 for distance and N6 for near, Intra Ocular Pressure (IOP) of < 21mmHg, and healthy anterior and posterior segments. All the participants underwent a comprehensive ophthalmic examination including SAP by using the Swedish Interactive Thresholding Algorithm (SITA) Standard (SS) 24-2 protocol in the Humphrey Field Analyser (HFA II—Carl Zeiss Meditec Inc. Dublin, CA). Reliability cut-offs were set according to the manufacturer's recommendations^13^. Maximum rate of reliability checks was not greater than 15% (for all three indices) in any of our participants. Whenever the reliability cut-off was crossed, perimetry was repeated after giving optimal rest breaks. Any participant presenting with a spherical ametropia ≥  ± 5.00 D and cylindrical ametropia of > − 2.00 D, nystagmus, strabismus, or ocular motility restrictions were excluded. Any presence of media opacity or cataract with a grade more than N2, C1, or P1 based on the Lens Opacification Classification System II (LOCS II) was excluded. Following the HFA, the participants underwent an EMP measurement. The experimental setup in the Netherlands and in India was maintained identically with respect to the hardware, technical specifications, customised test protocols, and room ambience.

### Apparatus and procedure

**Figure 1:**
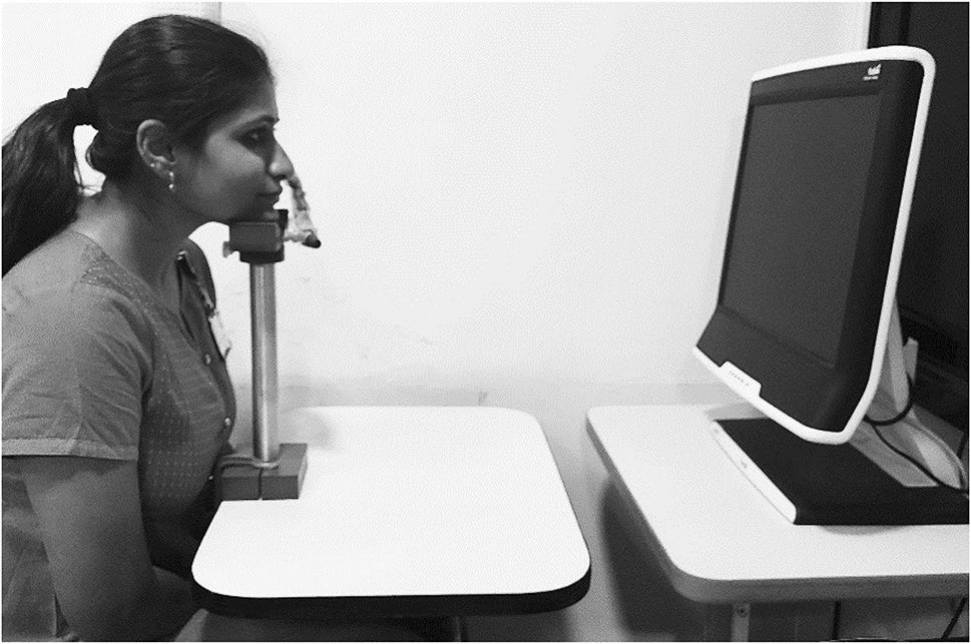
Eye movement perimetry (EMP) setup comprising a thin film transistor (TFT) display with an inbuilt Tobii T120 eye-tracking device. The chinrest was placed at a testing distance of 60 cm. (Participant shown is a volunteer from whom an additional informed consent was obtained for publishing this photograph).

The EMP test setup consisted of a 17-inch Thin Film Transistor (TFT) display with an inbuilt Tobii T120 eye tracking device with a refresh rate of 120 Hz (Fig. 1). The test was performed at a distance of 60 cm in a dimly illuminated room with noise kept at a minimum level. The EMP test started with an inbuilt calibration procedure where the participant was instructed to follow a red circular target that moved in a smooth pursuit fashion to nine cardinal gaze positions on the screen. The calibration procedure was repeated for the entire nine locations or specific locations in cases of insufficient gaze data samples or poor calibration accuracy. Upon passing the calibration test, the actual test was initiated.

The test was performed under monocular viewing conditions while the non-tested eye was covered with a black Polymethyl Methacrylate (PMMA) lens, to facilitate stable binocular gaze tracking. To be comparable with the clinical reference standard of testing the extent of the VF, the EMP test coordinates were kept analogous to the 24-2 SS protocol of the HFA. A total of 54 VF locations were tested, based on an overlap paradigm, by using Goldmann size III visual stimuli projected at four SI levels against a constant background. The basis for setting the background and the four levels of SI (background-to-stimulus contrast) was the RGB values. So fundamentally we used the functional notation of “unit RGB” that expresses a colour according to its red, green, and blue components in which each parameter defines the intensity of the colour with a value ranging between 0 and 1. This corresponded to four SI levels (192, 214, 249, and 276 cd/m^2^) against a background of 152 cd/m^2^. These stimuli had Weber contrasts of 26%, 41%, 64% and 82% which would correspond to the Weber contrasts for 31 dB, 29 dB, 27 dB, and 26 dB respectively on the HFA.

Each participant was instructed to look steadily at a fixation target and respond to a peripheral visual stimulus with an eye movement on detection followed by a re-fixation of the fixation target. To increase the extent of VF testing to ~ 54° horizontally and ~ 42° vertically on a flat screen, in addition to the central location, the fixation target was moved to four different eccentricities. The peripheral stimulus was projected for a maximum duration of 1200 ms with a gap of 0.2 s between the stimulus presentations. The interactive nature of the test protocol ensured a dynamic stimulus projection time window that was altered based on the eye movement response time. The total test duration was approximately 7–8 min per eye.

### Eye movement data processing

**Figure 2:**
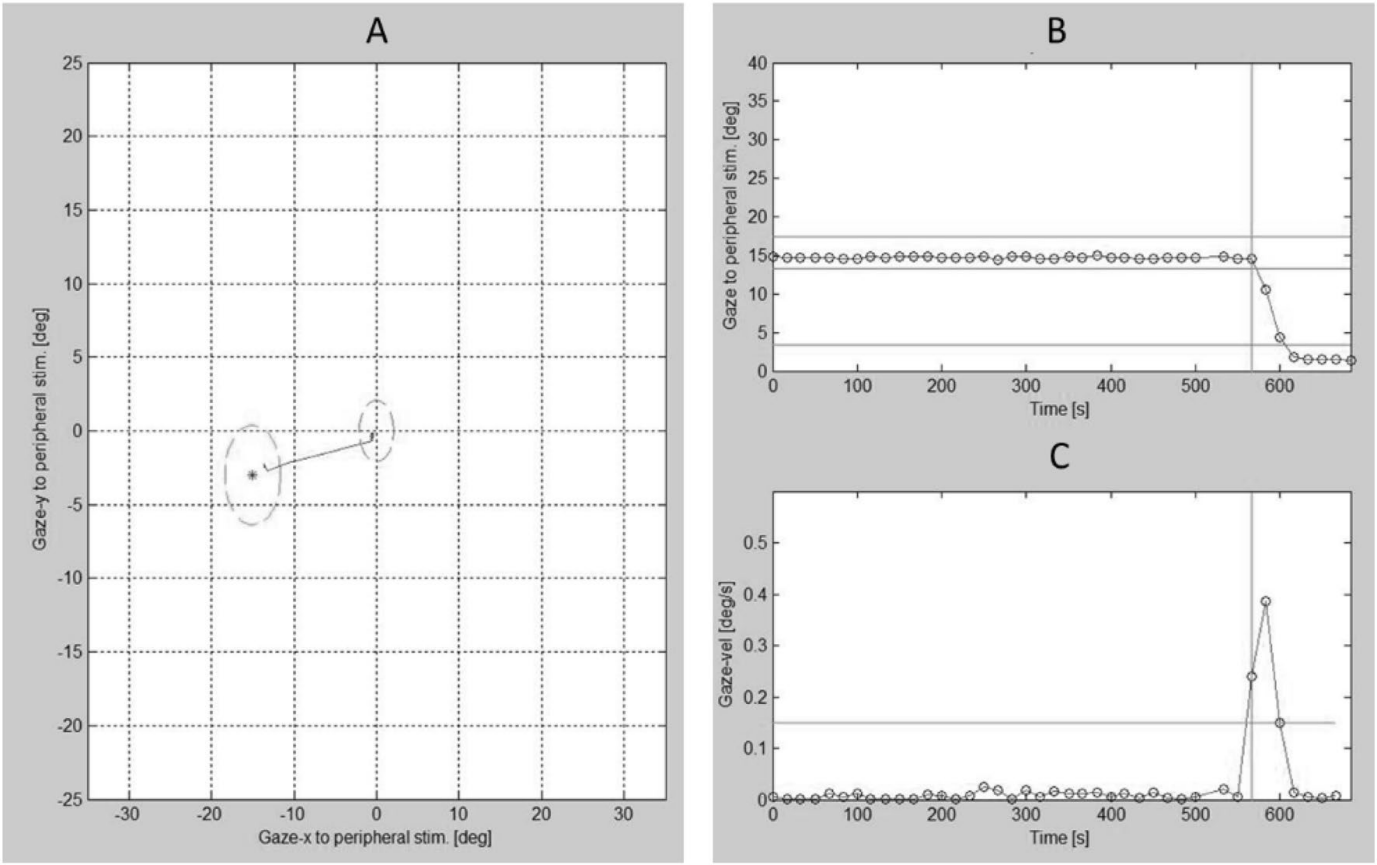
The customised Matlab window used for inspecting the trajectory and time course of a saccade initiated from the central fixation to a peripheral stimulus (**A**) with the corresponding gaze data (**B**) and gaze velocity signal (**C**).

The raw gaze data from 216 trials were visually inspected and further analysed by means of a custom-written Matlab program (Math Works, Natick, MA). Each tested location within each trial was classified as ‘seen’ when the three criteria was met: (a) a saccade was initiated towards the presented visual stimuli, (b) a saccade had covered > 50% of the distance from fixation towards the peripheral stimulus, and (c) a saccade was initiated within an angular disparity of  < 45 degrees between the direction of the primary SEM and the location of a peripheral stimulus. A tested location was labelled as ‘unseen’ if the above criteria were not satisfied. Trials in which SEM data was not available due to blinking or failure in detecting pupil centred corneal reflection, were labelled as ‘invalid’. From the trials categorised as ‘seen’, SRT values were calculated based on an ‘eye velocity’ criterion (when velocity exceeded 50°/s) and were defined as the time difference between the stimulus presentation and onset of the SEM towards the direction of the stimulus (Fig. 2).

### Statistical analysis

All statistical analyses were conducted with SPSS (Statistical Package for Social Sciences, Version 15, Chicago, IL, USA) by considering the responses obtained from the right eye. Demographic details and descriptive analyses were carried out for both ethnic cohorts. All tests were 2-tailed, and type I error was kept at a 5% level of significance. Age and SRT values among the ethnic groups were statistically compared using an independent t-test, and the proportion of gender was examined by means of the Chi-square test.

The study aimed to determine the influence of ethnic differences in combination with factors such as age, stimulus eccentricity, and SI. To investigate this, we used a Generalised Linear random effect Mixed Model analysis (GLMM). This method investigated the interactions and effects (random) within the factors while adjusting the SRTs for each independent factor. The GLMM included the analysis of variance for one dependent variable (SRT) and four independent factors (ethnicity, age group, stimulus eccentricity, and SI). For this, the participants were divided into two ethnic groups i.e. Indian and Dutch, and five age groups: 20–29, 30–39, 40–49, 50–59, and 60 years and above. The tested VF was divided into five distinct zones based on the stimulus eccentricities i.e. 4°, 11°, 16°, 22° and, 27°. Also, we had responses obtained at four levels of SI. The interaction between these independent factors with SRT was added to the model by using the equation: ethnicity × age group × stimulus eccentricity × SI. The output of a GLMM univariate estimated the Mean Difference (MD) and its corresponding Confidence Interval (CI) and a significant interaction were further interpreted by subsequent pairwise comparisons (post hoc analysis). Based on the output from the post hoc analysis, we pre-decided that only if a > 10% (proportion) of the pair-wise estimates (100 comparisons in total) were significant we would consider ethnicity to have a substantial influence on SRT. If in case of a prominent effect (> 10%) then we would conclude that the differences should be accounted for while implementing EMP in a diverse set of populations drawn from different parts of the world.

### Ethical considerations

The study adhered to the declaration of Helsinki for research involving human participants and the study protocol and procedures were reviewed and approved by the Institutional Review Board of Vision Research Foundation, Chennai, India, the Medical Ethics Committee of Erasmus University Medical Centre, and the Rotterdam eye hospital, Rotterdam, The Netherlands. Written informed consent was obtained from all the participants before enrolment and initiation of procedures.

## Results

The study recruited 149 healthy participants including 86 Indian and 63 Dutch individuals whose demographic details are summarised in Table 1. The spread of participants in the different age groups showed relatively larger numbers in the younger age group among Indians whereas among the Dutch the numbers were larger in the oldest age group (≥ 60 years).

**Table 1:** Demographics and data summary of the healthy adults from the Indian and Dutch ethnic groups.

Parameters	Indian (n = 86)	Dutch (n = 63)	p-values
Age range (years)	20–75	20–85	NA
Mean age (SD) in years	48 (13)	50 (12)	0.34^a^
Age groups: range (n)	20–29 (22)	20–29 (11)	NA
	30–39 (18)	30–39 (8)	
	40–49 (20)	40–49 (8)	
	50–59 (15)	50–59 (8)	
	≥ 60 (11)	≥ 60 (28)	
Gender: n (%)			0.87^b^
Male	45 (52)	33 (52)	
Female	41 (48)	30 (48)	

*NA* Not Applicable.

^a^Independent t-test.

^b^Chi-square test.

### Comparison of healthy Indian and Dutch adults: EMP performance and SRT

Eye movement performance described based on the percentage of ‘seen’ and ‘unseen’ responses was found to be comparable between the ethnic cohorts. The percentage of ‘seen’ responses in both the cohorts ranged from 90 to 70% which showed a trend of decreasing percentage of ‘seen’ responses with an increase in age. The overall mean SRT (SD) values of the Indian cohort was not statistically significantly different when compared to the Dutch cohort (p = 0.21, Independent t-test) which were 417 (157) ms and 426 (147) ms respectively. A random effect GLMM analysis performed with SRT as a dependent variable revealed an overall statistically significant interaction (p < 0.001) between SRT and the independent factors, ethnicity, age group, stimulus eccentricity, and SI (Table 2).

**Table 2:** The random effect generalised linear univariate model of the individual factors used to analyse the ethnic differences in SRT.

Source	df	Mean square	F	p-values^a^
Corrected model	99	305,071.61	16.03	< 0.001
Intercept	1	537,866,801.9	28,263.96	< 0.001
Ethnicity*age (5 groups) *Eccentricity (5 zones) *SI (4 levels)	94	170,211.89	8.94	< 0.001

*df* Degrees of Freedom, *SI* Stimulus Intensity.

^a^General Linear Mixed Model analysis.

Next, a comprehensive overview of the pairwise comparisons (100 in total) for the significant main interactions of the factor levels (ethnicity, age, stimulus eccentricity, and intensities) with SRT differences between the Indian and Dutch ethnic cohorts was described (Table 3).

**Table 3:** GLMM Univariate output showing the pairwise contrast estimates (6%, 6/100) between the levels of factors (ethnicity, age, stimulus eccentricity, and SI).

Ethnicity (Indian-Dutch)		Age groups (years)	Eccentricity	SI (cd/m^2^)	Mdiff(ms)	p-values	95% CI	
							Lower	Upper
Indian	Dutch	≥ 60	11°	214	64	0.0002	31	97
Indian	Dutch		16°		60	0.0003	29	91
Indian	Dutch		22°		49	0.0002	19	79
Indian	Dutch	20–29	22°	276	− 50	< 0.0001	− 71	− 28
Indian	Dutch	40–49			− 62	< 0.0001	− 89	− 35
Indian	Dutch	50–59			− 63	< 0.0001	− 93	− 33

*Mdiff* Mean Differences in SRT (ms), *p-values* Adjusted p-values with post hoc (Bonferroni correction), *SI* Stimulus Intensities, *CI* Confidence Intervals.

**Figure 3:**
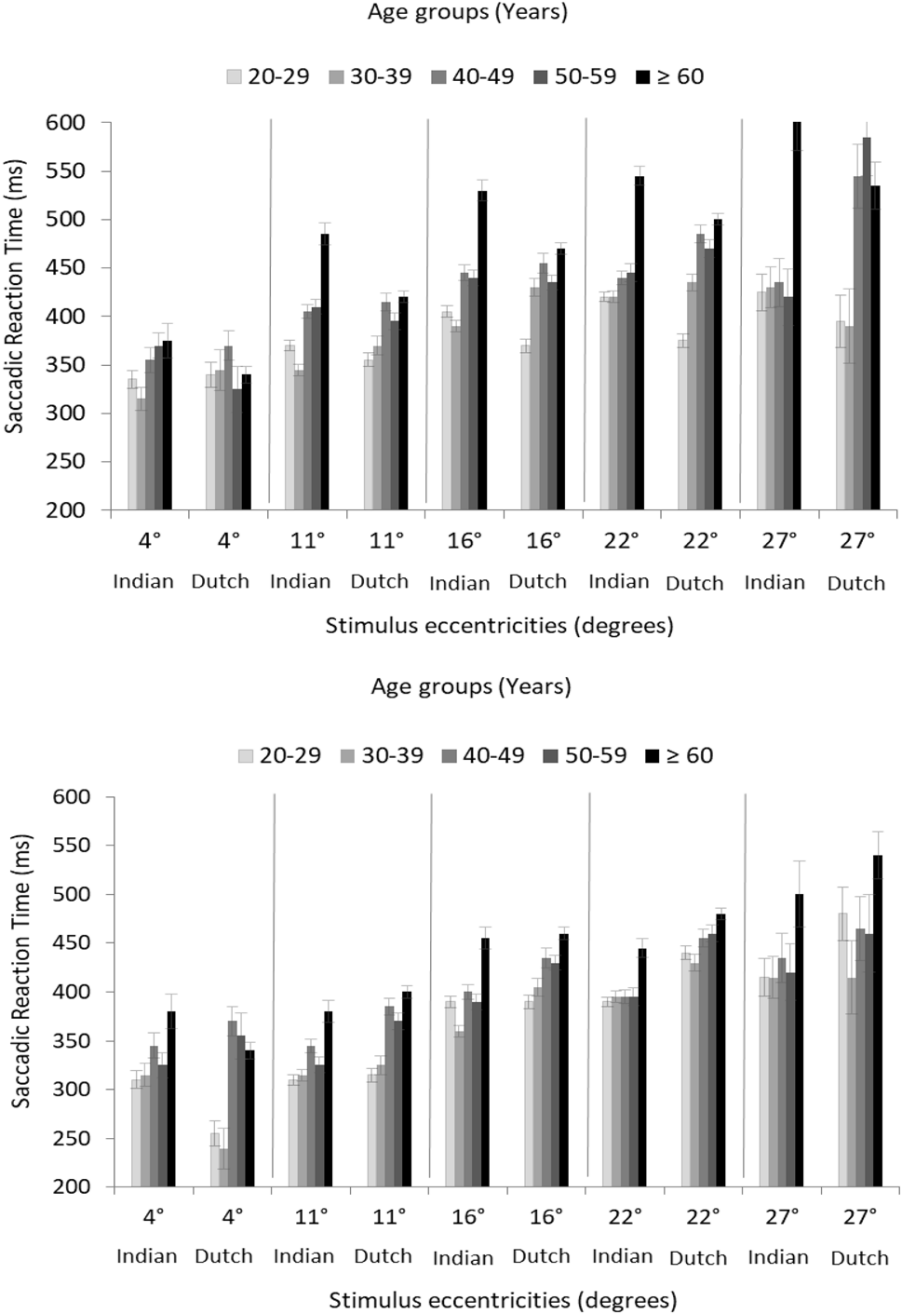
Cluster bar graphs displaying the mean SRT (ms) values among the healthy adults from the Indian and Dutch ethnic cohorts for the stimulus intensities 214 cd/m^2^ (top panel) and 276 cd/m^2^ (bottom panel) across the stimulus eccentricities and all the five age groups. Error bars represent the Standard Error.

The Indian ethnic cohort, aged ≥ 60 years exhibited statistically significantly delayed SRTs at 11°, 16°, and 22° eccentricities for the 214 cd/m^2^ intensity level when compared to their Dutch counterparts (p < 0.001). However, the Indian cohort aged below 60 years showed statistically significantly faster SRTs at 22° eccentricity for the 276 cd/ m^2^ intensity level. A statistically significant delay in SRT as a function of increasing age and stimulus eccentricity, and decreasing SI was found in both ethnic cohorts (Fig. 3).

## Discussion

Previous studies have reported how differences in culture influence the way individuals view and encode visual scenes specifically differences in the pattern of saccades and fixations. Hence, it seemed pertinent to do this study while introducing an alternative mode of perimetry, based on saccadic eye movements, among diverse ethnic cohorts. Genetic studies have made it clear that there is no biological basis for race, which is a social construct, so the appropriate analysis is to compare people from different countries as is done in this study. Here, we answer the question of whether people drawn from distinct parts of the world have significant differences in SRT values.

### Comparison of healthy Indian and Dutch adults: EMP performance and SRT

In summary, in both the ethnic cohorts, a statistically significant delay in SRT was found as a function of increasing age and stimulus eccentricity, and decreasing SI (Fig. 3). The pairwise comparison (post hoc of the GLMM) showed that the Indian ethnic cohort (aged ≥ 60 years) exhibited statistically significantly delayed SRTs for the 214 cd/m^2^ intensity level when compared to their Dutch counterparts (but only at 11°, 16°, and 22° eccentricity) whereas those aged below 60 years showed statistically significantly faster SRTs (at 22° eccentricity) for the 276 cd/m^2^ intensity level. Since these observed differences were found in only specific stimulus eccentricities we don’t think it is generalizable to confidently state that older Indians are delayed in terms of SRT when compared to their Dutch counterparts. This difference might even be due to the unequal proportion of the sample in these age groups. Even if both the ethnic cohorts showed a statistically significant delay in SRT with progressing age, remarkably, the Indian cohort showed a higher magnitude of SRT shift when compared to the Dutch. Since cataract often co-exist with conditions like glaucoma causing an overall decline in visual field sensitivity, caution was taken while setting our inclusion criteria to permit the enrolment of participants with the best corrected visual acuity of ≥ 20/40 for distance and N6 for near and healthy anterior and posterior segments with no clinically significant cataract. This aided us in controlling the possible confounding effect of cataract on SRT^14^. Therefore, the higher magnitude of SRT delay found among elderly Indians (≥ 60 years) can’t be attributed to either cataract-related changes or actual ethnic differences. Instead, the possible explanation is the low number of Indian participants in that particular age bin (n = 11). Although efforts were taken to enroll an equal proportion of individuals in each age group identifying visually healthy adults older than 60 years specifically from the Indian side (Southern India)^15^ was challenging given the high rate of un-operated cataract especially considering the stringent inclusion criteria set for age-related changes in the optical media and ocular surface. Previous studies have reported Indians to exhibit an early onset of age-related ocular changes including early senile changes of ocular media^15,16^ or premature presbyopia etc.^17^. Hence, it might be worthwhile investigating the rate of age-related deficits in contrast detection and control over SEM generating networks between different ethnic cohorts. An exploration of its association with pre-retinal factors such as pupil size and transmission losses of the ocular media or neural losses in the visual pathway might also add relevance^18^.

Several previous literatures that investigated eye movement tasks concerning visual attention generally neither reported nor considered ethnicity/cultural variability as a confounding factor that might influence the outcome measures. However, a few studies have reported the similarities/dissimilarities in eye movement behaviour between culturally diverse participants drawn from different human populations^3,19,20^. A study that compared the global versus local visual attention in a cohort of East Asians and Caucasian Westerners showed that Asians are markedly inclined to a global attentional processing style^19^. On the other hand, a study that dealt with eye movement evaluation during face recognition tasks demonstrated that a cohort of British-born Chinese equated well with the approaches exhibited by East Asian participants^20^. Another study that investigated the differences between Chinese and American participants while performing six different tasks found no significant differences in the fixation duration in the visual search and the scene perception tasks between the two cultural cohorts. The study suggested that the observation of differences in the characteristics of eye movement across the ethnic cohorts might be task-dependent and cannot be generalized^3^. Our study also showed (at eccentricity levels of 11°, 16°, and 22°) the older Indians, ≥ 60 years with a significantly delayed SRT, and the trend was found to be inverted when it comes to the groups < 60 years of age. This transition cannot be confirmed due to the inclusion of fewer participants in the age group above 60 years but could be further investigated to know if it is attributable to the variations in the spatio-temporal perception modulated by the exposure to environmental dynamics.

Even though specific measures have been taken to ensure identical test setups with respect to instrumentation and experiment room, the interfacility variability between the institutions might have an impact on the performance of the study participants, which could not be precisely controlled. Furthermore, the ethnicity of the participants was ‘self-identified/reported ethnicity’ and not on specific anthropologically based criteria.

## Conclusions

In both ethnic cohorts, a statistically significant delay in SRT was found as a function of increasing age, stimulus eccentricity, and decreasing stimulus intensity. Although the Generalized Linear Mixed Model analysis revealed an overall statistically significant influence of ethnicity and interaction between the tested factors, the post hoc estimate showed statistical significance in only a minor proportion of pair-wise comparisons. Hence, the ethnic-specific differences need not be certainly accounted for while implementing EMP in a diverse set of populations drawn from different parts of the world instead a collective database might serve the purpose.

## Data Availability

The datasets generated and/or analysed during the current study are not publicly available because the data serves as the normative database for the proposed prototype, but are available from the corresponding author on reasonable request.
